# The assessment of pentraxin 3: a diagnostic and prognostic biomarker in lower respiratory tract infections in children

**DOI:** 10.1186/s13052-024-01735-5

**Published:** 2024-09-18

**Authors:** Shaimaa Madkour, Mona Gamal Mostafa, Huda El-Kady

**Affiliations:** 1 Department of Pediatrics, Faculty of Medicine, Fayoum University, Fayoum, Egypt; 2 Department of clinical and chemical pathology, Faculty of Medicine, Fayoum University, Fayoum, Egypt

**Keywords:** Pentraxin 3, Children, Lower respiratory infections, C-reactive protein

## Abstract

**Background:**

Pentraxin 3 (PTX3) is an acute-phase reactant that is elevated in the plasma during inflammatory responses. We aimed to evaluate the utility of PTX3 as a clinical marker in children with lower respiratory tract infections (LRTIs) and the association between PTX3 and LRTIs severity.

**Methods:**

We included 60 patients admitted to Fayoum University Hospital with LRTIs fulfilling the WHO criteria for diagnosing LRTIs. We collected data on peak temperature, respiratory rate, heart rate, oxygen saturation upon admission, and length of hospital stay. The complete blood count (CBC), C-reactive protein (CRP) level, and PTX3 were measured upon admission.

**Results:**

PTX3 levels were significantly correlated with peak temperature, duration of hospital stay, the Pediatric Respiratory Severity Score (PRESS), total leucocytic count (TLC), CRP, and blood cultures.

**Conclusion:**

PTX-3 represented the severity of the disease and predicted the prognosis. Pentraxin levels demonstrate a statistically significant sensitivity of (93.3%) and a specificity of (70%) at the cut-off value (of 8.84) with an area under the curve (90.7%) in the diagnosis of LRTIs.

## Background

Worldwide, lower respiratory tract infections (LRTIs), especially in children, are the leading causes of morbidity, hospitalization, and mortality [1]. For an effective course of treatment, an early diagnosis and assessment of the disease’s severity are essential. For this reason, a variety of inflammatory markers have been established, including procalcitonin (PCT) levels, CRP levels, and erythrocyte sedimentation rate (ESR) [2].

Acute-phase inflammatory mediator pentraxin 3 (PTX3) is formed at the infection site and can be tested within a few hours. Endothelial cells, epithelial cells, and leukocytes can generate PTX3 in the lungs if properly activated [3].

It has been demonstrated that PTX3 can identify various infectious organisms, including bacteria, viruses, and fungi, and can improve their removal during experimental pneumonia. This is mostly accomplished by controlling neutrophil recruitment [4]. Humans with pleural fluid PTX3 measurement have improved parapneumonic effusion (PPE) discrimination, circulating PTX3 is increased in the most severe types of ventilator-associated pneumonia (VAP) and community-acquired pneumonia (CAP) and plasma PTX3 levels correlate with clinical severity in numerous infectious illnesses [5].

VAP can be defined as pneumonia that develops for more than 48 h following the start of mechanical ventilation and endotracheal intubation. For Intensive care unit (ICU) staff, a definitive VAP diagnosis remains difficult. According to the CDC guidelines, leukocytosis, fever, purulent lung secretions, and new pulmonary infiltrates on chest X-rays should all be present for the clinical VAP diagnosis to be established [6]. A reliable standard for diagnosing VAP is a bacterial culture of bronchoalveolar lavage, although results often take two to three days to obtain [7].

This study was designed to assess the value of PTX3 in the diagnosis of LRTIs cases as well as its ability to predict severity.

## Methods

### Study design

The present work was carried out at the Fayoum University Pediatric Hospital as a case-control study between March 2023 and March 2024.

### Sample size

was determined using the Institute of Experimental Psychology, Heinrich Heine University, Dusseldorf, Germany’s G-Power©ฏ software, version 3.1.7. There were 120 patients in the entire sample. A two-sided (two-tails) type I error of 0.05 and a power of 80% with an effect size of 0.353 were based on the findings of other studies. The sample size was raised by 10% to account for incomplete or missing data.

### Inclusion criteria

Using the WHO criteria for diagnosis of LRTIs, which include fever, cough, rapid breathing rate relative to age (specified as ≥ 60 breaths/min for infants under 2 months, ≥ 50 breaths/min for those 2 to 11 months, ≥ 40 breaths/min for those 12 to 59 months, and > 20 breaths/min for those older than 59 months.), chest in-drawing, and rhonchi or crepitations on auscultation [8–10]. The final sample size comprised 60 patients aged beyond 1 month to 6 years with LRTIs as the case group and 60 healthy children matched for age and sex as the control group. The pediatric ward, intermediate care, and pediatric intensive care unit (PICU) of Fayoum University Hospital in Fayoum, Egypt, were used to select children with LRTIs.

LRTIs were categorized as follows: ▶ Pneumonia: history and clinical evidence of fever or respiratory symptoms, together with pulmonary infiltration on a chest x-ray (CXR) ▶ Acute bronchiolitis: cough, dyspnea, tachypnea, wheezing, crepitations and pulmonary hyperinflation on CXR, after upper respiratory tract infection in children under two years old [11]. ▶PPE was characterized by a collection of pleural effusions detected by chest ultrasonography or radiograph in conjunction with underlying bacterial pneumonia [12].

### Exclusion criteria

Children were not included if they had severe malnutrition, other concurrent infections, or underlying chronic disease (such as chronic respiratory diseases like cystic fibrosis and primary ciliary dyskinesia, hemoglobinopathy, immunological deficiencies, congenital heart disease, psychomotor retardation, neurological conditions that progress over time).

### Methodology

Every patient received a thorough workup, including a full medical history and physical examination. Upon admission, we recorded clinical data on peak fever, respiratory rate, heart rate, oxygen saturation, presenting symptoms, and chest auscultatory findings.

Five components were used to calculate the Pediatric Respiratory Severity Score (PRESS): respiratory rate, wheezing, accessory muscle use, oxygen saturation, and feeding problems. For every parameter, there is a score of 0 if it is absent and 1 if it is present. LRTIs are categorized as mild (score 0 to 1), moderate (score 2 to 3), and severe (score 4 to 5) based on the total of these factors [13].

The chest radiograph was done. The laboratory workup for all patients included CBC with differential, CRP levels, an arterial blood gas analysis (ABG), and a blood culture with sensitivity. A sputum culture was performed if the patient showed no clinical improvement after one week of treatment.

### Measurement of serum pentraxin 3 level

Venous blood samples were collected from all subjects into a plain tube, and the serum samples were stored at -20 °C. Serum samples were tested for PTX3 using a human PTX3 enzyme-linked immunosorbent assay (ELISA) kit (Bioassay Technology Lab, Cat. No. E1938Hu, China), using a double-antibody sandwich ELISA. The optical densities (OD) of samples and standards were measured at 450 nm wavelength by a microplate reader. Lastly, the standard curve linear regression equation was calculated based on the concentration of the standards and the corresponding OD values. From this, the concentration of the samples was determined.

### Statistical analysis

The Statistical Package of Social Science (SPSS) software version 22 (SPSS Inc., Chicago, IL, USA) and Windows 7 were used for data analysis. Standard deviations are used to measure the dispersion of quantitative parametric data, whereas arithmetic means are used to measure central tendency. Qualitative data is expressed as percentages and numbers in simple descriptive analysis. The student t-test, one-way ANOVA-F test, Mann-Whitney U test, and Kruskal-Wallis H test are used to compare quantitative data. Applying the Chi-square test to qualitative data. To assess the link between quantitative variables, utilize the bivariate Pearson correlation test. The relationship between categorical dependent and independent variables and predictive risk factors was examined using a regression test. By utilizing the “Receiver Operating Characteristic” (ROC) curve, test for specificity and sensitivity. P-values were considered statistically significant if they were less than 0.05.

## Results

In total, 120 subjects were included in the study (60 healthy children as the control group and 60 LRTI patients as the study group). Of the 60 LRTI patients, 43.3% were males and 56.7% were females, with a mean age of 18.2 ± 22.6 months. Cases and controls showed no statistically significant difference in age or sex. Demographic characteristics are summarized in Table 1.

**Table 1:** Comparisons of demographic characters in different study groups

Variables	Cases (N = 60)		Control (N = 60)		P-value
**Age (months)**					
Mean ± SD	18.2 ± 22.6		24.3 ± 23.5		0.16
**Sex**					
Male	26	43.3%	28	46.7%	0.85
Female	34	56.7%	32	53.3%	

SD: Standard deviation

In all, 36.7% of cases were admitted to the PICU and 33.3% to the intermediate care. All cases complained of dyspnea, followed by cough (93.3%) and poor food intake (86.7%). By clinical examination, cases suffered from fever (median 38.5), a high respiratory rate (median 60 breaths/min), and a high heart rate (median 116 beats/minute). Chest examination revealed crepitations (93.3%) and diminished air entry (76.7%). Clinical characteristics are illustrated in Table 2.

**Table 2:** Medical history and clinical features among cases group

Variables (n = 60)	Description	
**Site of admission**	**N**	**%**
Ward	18	30%
PICU	22	36.7%
Intermediate care	20	33.3%
**Presentation**	**N**	**%**
Dyspnea	60	100%
Cough	56	93.3%
Poor oral intake	52	86.7%
Rhinorrhea	22	36.7%
Vomiting	22	36.7%
**General examination**	**Median**	**(Range)**
Duration of fever (days)	4.5	(1–14)
Peak body temperature (°C)	38.3	(37–40)
Respiratory rate (breaths/min)	60	(44–80)
Heart rate (beats/min)	116	(98–136)
PRESS	3	(0–5)
O_2_ saturation (%)	90	(70–96)
Hospital stay duration (days)	16	(4–27)
**Chest Examination**	**N**	**%**
Crepitations	56	93.3%
Diminished air entry	46	76.7%
Wheezes	40	66.7%
**Respiratory failure**	18	30%
**Diagnosis**	**N**	%
Pneumonia	38	63.3%
Bronchiolitis	6	10%
VAP	10	16.7%
PPE	6	10%
**Management**	**N**	**%**
O_2_ Support	60	100%
Mechanical ventilation	18	30%
IV fluid	52	86.7%
**Outcome**	N	**%**
Discharged	58	96.7%
Died	2	3.3%

PICU: Pediatric intensive care unit, VAP: Ventilator-associated pneumonia, IV: Intravenous, PRESS: Pediatric Respiratory Severity Score, PPE: parapneumonic effusion

Case management regimens revealed that 100% received oxygen therapy, 30% required mechanical ventilation, and 86.7% needed intravenous fluids. The majority of cases (96.7%) were fully recovered and discharged from the hospital, while 3.33% died. Table 2 presents management and outcome data.

Our cases had a high level of CRP (median 31 mg/L), an elevated TLC count with a median of 16 (10^3^/ mm^3^), mild anemia (9.4 g/dl ± 1.4), and a normal platelet count with a median of 368.5 (10^3^/mm3). Blood culture was done for all cases, with 30% of cases containing bacterial growth and 22.2% for each of Klebsiella, pseudomonas, and MRSA. The majority of the growth was resistant to different types of antibiotics. Sputum culture was done in 63.3% of cases, with 13.8% of them showing bacterial growth and 50% of them revealing pseudomonas. The majority of growth showed resistance to different types of antibiotics. Laboratory findings are demonstrated in Table 3.

**Table 3:** Laboratory findings among cases group

Variables (n = 60)	Description	
	Median	(Range)
**CRP** (mg/L)	31	(5-207)
**CBC**		
TLC (10^3^/mm^3^)	16	(6.8–38.1)
Hemoglobin (g/dl)	9.1	(7-12.3)
Platelet (10^3^/mm^3^)	368.5	(37–895)
**ABG measures**		
PH	7.3	(6.9–7.5)
PO_2_ (mmHg)	84.5	(60–93)
HCO_3_ (mEq/L)	21	(15–31)
PCO_2_ (mmHg)	46	(21–69)
**Blood culture**	**N**	**%**
No growth	42	70%
Growth	18	30%
**Type of growth** (***n*** = 18)	**N**	**%**
Klebsiella	4	22.2%
Pseudomonas	4	22.2%
CoNS	2	11.1%
MRSA	4	22.2%
Candida non albicans	2	11.1%
Acinetobacter	2	11.1%
**Sputum culture**	**N**	**%**
No growth	30	50%
Growth	8	13.3%
Not done	22	36.7%
**Type of growth** (***n*** = 8)	N	**%**
Klebsiella	2	25%
Pseudomonas	4	50%
Acinetobacter	2	25%

CRP: C-reactive protein, CBC: Complete blood count, TLC: Total Leucocytic count, CoNS: Coagulase-negative staphylococci, MRSA: Methicillin-resistant staphylococcus aureus

**Fig. 1:**
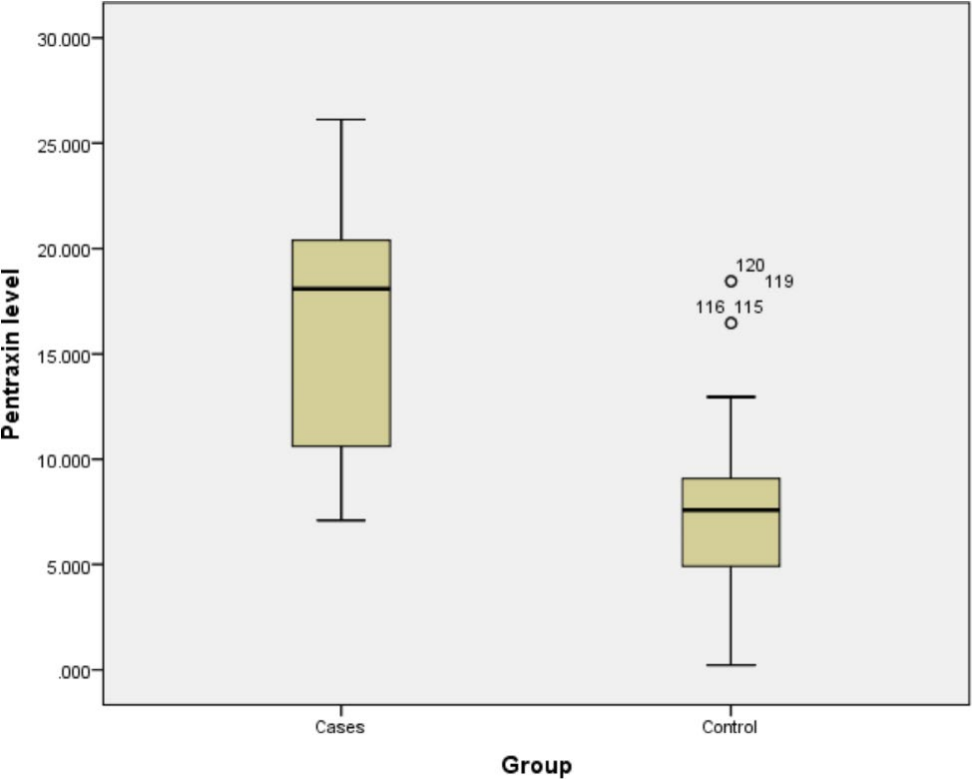
Comparisons of PTX3 level in different study groups

Cases had statistically significantly higher mean serum levels of pentraxin 3 (16.7 ± 5.7) than controls (7.6 ± 3.9), with a p-value < 0.001 (Fig. 1).

Serum PTX3 concentrations were significantly higher in patients admitted to the PICU, patients with respiratory failure, VAP, and patients who required mechanical ventilation in management. On the other hand, there was no statistically significant difference in PTX3 level with a p-value > 0.05 concerning sex, outcome, or IV fluid in their management (Table 4).

**Table 4:** Comparisons of PTX3 level in different clinical data and culture results among cases

Variable	PTX3 level		P-value
	Mean	SD	
**Sex**			
Male	11.8	5.7	0.54
Female	12.5	7.4	
**Site of admission**			
Ward	17.4	5.5	0.01*
PICU	18.8	5.1	
Intermediate care	13.8	5.4	
**Respiratory Failure**			
No	15.5	5.6	0.007*
Yes	19.7	4.8	
**Diagnosis**			
Pneumonia	16.6	6.1	0.04*
Bronchiolitis	13.05	4.8	
VAP	20.5	2.6	
PPE	14.8	4.3	
**Management**	**No**	**Yes**	
Mechanical ventilation	15.5 ± 5.6	19.7 ± 4.8	0.006*
IV fluid	17.1 ± 4.8	16.7 ± 5.8	0.82
**Outcome**	**Mean**	**SD**	
Discharged	16.6	5.7	0.38
Died	20.2	0	
**Blood culture**	**Mean**	**SD**	
No growth	15.7	5.6	0.04*
Growth	18.9	5.2	
**Type of blood growth (n = 18)**	**Mean**	**SD**	
Klebsiella	18.5	8.7	0.17
Pseudomonas	19.2	0.14	
CONS	26.1	0	
MRSA	20.7	0.58	
Candida non albicans	14.1	0	
Acinetobacter	13.2	0	
**Sputum culture**			
No growth	15.03	5.7	0.34
Growth	17.2	5.5	
**Type of Sputum growth (n = 8)**			
Klebsiella	11.05	0	0.04*
Pseudomonas	21.9	3.3	
Acinetobacter	14.1	0	

CoNS: Coagulase-negative staphylococci, MRSA: Methicillin-resistant staphylococcus aureus, PPE: parapneumonic effusion, *: Significant

Cases with positive bacterial blood cultures had statistically significant increased levels of PTX3 (p-value = 0.04), regardless of the kind of organism in the growth. In contrast, there was no statistically significant difference in PTX3 levels (p-value > 0.05) between sputum cultures. However, sputum cultures for Pseudomonas growth revealed a higher level of PTX3 than other types of pathogens (Table 4). Increasing hospital stay duration, PRESS score, CRP, TLC, HCO_3_, PCO_2_, and body temperature levels and decreasing hemoglobin, PH, PO2, and oxygen saturation levels were significantly correlated with increased pentraxin 3 levels among patients (p-value < 0.05) as indicated in Table 5.

**Table 5:** Correlation of PTX3 level in different clinical and investigation data among cases

Variables	PTX3 level	
	R	P-value
**Clinical features**		
Age (months)	-0.06	0.54
Duration of fever	0.15	0.24
Hospital stays	**0.57**	0.001*
Peak body temperature	**0.45**	0.001*
Respiratory rate	0.07	0.58
Heart rate	-0.11	0.37
O_2_ saturation	**-0.40**	0.001*
**PRESS**	**0.32**	0.01*
**Investigations**		
**CRP**	**0.39**	0.003*
**CBC**		
TLC	**0.30**	0.01*
Hemoglobin	**-0.30**	0.02*
Platelets	0.07	0.59
**ABG**		
PH	**-0.34**	0.01*
PO_2_	**-0.35**	0.006*
PCO_2_	**0.32**	0.01*
HCO_3_	**0.28**	0.03*

CRP: C-reactive protein, CBC: Complete blood count, TLC: Total Leucocytic count, ABG: Arterial blood gas analysis, PRESS: Pediatric Respiratory Severity Score, *: Significant

The linear regression model analysis illustrated a statistically significant prediction effect of pentraxin 3 in predicting the duration of hospital stay with a p-value of 0.011 (Table 6).

**Table 6:** Linear regression analysis to determine the power of different variables in predicting hospital stay duration

Model	Unstandardized Coefficients		Standardized Coefficients	T	Sig.
	B	Std. Error	Beta		
(Constant)	7.500	3.053		2.457	0.017
PTX3 8.84	8.286	3.160	0.326	2.622	0.011*

*: Significant

The logistic regression model analysis demonstrated a statistically significant prediction effect of pentraxin 3 in the estimation of mechanical ventilation requirement with a p-value of 0.012 (Table 7).

**Table 7:** Logistic regression analysis to determine the power of different variables in predicting the need for mechanical ventilation in cases

Model	B	S.E.	Wald	Df	Sig.	Exp(B)	95% C.I.for EXP(B)	
							Lower	Upper
Pentraxin 3 Constant	0.151	0.060	6.316	1	0.012*	1.162	1.034	1.307
	-3.507	1.150	9.296	1	0.002*	0.030		

*: Significant, C.I: confidence interval

**Fig. 2:**
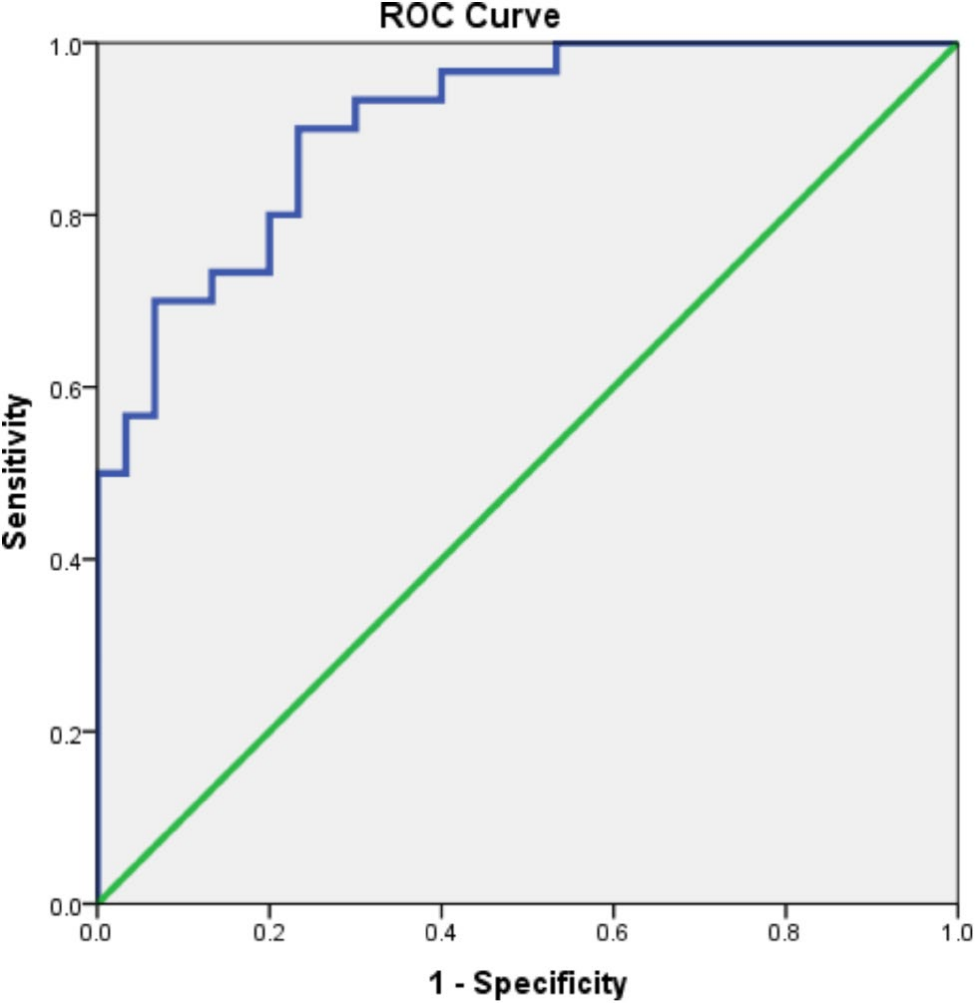
ROC curve for PTX3 level in the diagnosis of cases

Pentraxin 3 level presented a statistically significant sensitivity of 93.3% and a specificity of 70% at a cut-off value of 8.84 ng/ml with an AUC of 90.7%, PPV of 75.7%, and NPV of 91.3% in the diagnosis of cases compared to controls (Table 8; Fig. 2).

**Table 8:** Sensitivity and specificity of Pentraxin level in the diagnosis of cases

Variable	Sensitivity	Specificity	AUC	PPV	NPV	Cut off point	p-value (95% CI)
Pentraxin level	93.3%	70%	90.7%	75.7%	91.3%	8.84	< 0.001 (0.857–0.956)

AUC: Area under curve, PPV: positive predictive value, NPP: Negative predictive value, CI: Confidence interval

## Discussion

Infections in the respiratory tract continue to be the most frequent cause of medical visits [14]. To begin the proper course of therapy, a quick and precise diagnosis of respiratory tract infections is essential [15]. An essential part of innate immunity linked to sepsis is pentraxin 3, the first member of the long pentraxin subfamily to be discovered [16]. Pentraxin 3 recognizes microbes, facilitates pathogen detection, and controls complement activation, which is crucial in the early phases of inflammation [17].

By demonstrating a positive correlation with the peak temperature, length of hospital stays, PRESS score, respiratory failure, need for mechanical ventilation, and PICU admission, we found out that the level of PTX3 represents the severity of the disease in children with LRTI. This is in line with earlier research on the use of the PTX3 level to predict the severity of infectious diseases [18]. When triggered, a systemic inflammatory response carried on by severe pneumonia may lead the body to release more PTX3 into the blood. Furthermore, there is a protracted treatment course, a gradual symptom remission, and a protracted half-life of PTX3 in the body [19].

Nevertheless, PTX3 levels showed no significant correlation with respiratory rate, or heart rate in agreement with Kim et al. [8].

In line with ZHOU et al. [20], we showed that the PTX3 level was positively correlated with PCO_2_ and negatively correlated with oxygen saturation and PaO_2_.

Unlike Kim et al. [8], PTX3 indicated a strong correlation with total TLC in the current study.

Furthermore, we focused on the relationship between infectious pathogens and PTX3 levels. Regardless of the kind of organisms detected in the growth, those with positive blood culture growth indicate a statistically significant higher level of PTX3. However, there is no statistically significant difference in the level of PTX3 in the outcomes of sputum cultures. However, sputum cultures for Pseudomonas growth cases demonstrate a greater level of PTX3 compared to other organism types. The findings of this study were supported by previous literature, which suggested that patients with different pathogen infections had variable PTX3 levels [21], However, further studies also reported that there were no significant differences between patients with and without infections [22].

In line with Elmahalawy et al.‘s [23] findings that PTX3 is specific in the diagnosis of VAP, our analysis revealed a statistically significant greater level of PTX3 among cases identified as VAP.

In contrast to Ozsu et al.‘s [24] finding that PTX-3 levels were significantly higher in pleural effusions arising from strong inflammation, such as PPE, our analysis revealed that the level of PTX3 was higher in pneumonia than in PPE. Our differences can be explained by the following: (1) we measured PTX3 in serum rather than pleural fluid; (2) there were only 6 patients with parapneumonic effusions compared to 38 patients with pneumonia; and (3) we measured PTX3 at the time of admission in the early stages of pleural effusion and the results of their pleural fluid cultures were negative.

In accordance with Siljan et al. [25], there was a strong association between CRP and PTX3 in our study’s subjects. Unlike CRP, PTX3 is generated at the infection site by a variety of cell types, such as monocytes and neutrophils, in response to inflammatory triggers (such as cytokines or microbial moieties) [26].

The linear regression model analysis showed that pentraxin 3 predicted the duration of hospital stay, in contrast to Kim et al. [8], who found that PTX 3 failed to predict the prognosis.

With a sensitivity of 93.3% and a specificity of 70%, the PTX3 level in LRTI patients in this clinical study was significantly greater than that in healthy controls. Elmahalawy et al.‘s study [23] revealed sensitivity and specificity of 96.8% and 100%, respectively. Pentraxin 3 had a 50.9% diagnostic specificity in the Thulborn et al. study findings [27].

A summary of the whole test’s accuracy is presented by the ROC curve. Areas under the ROC curve with values between 0.50 and 0.70 denoted low accuracy; 0.70 and 0.90 indicated moderate accuracy; and values over 0.90 indicated excellent accuracy [28]. The area under the ROC curve in the current investigation was 90.7, demonstrating pentraxin 3’s high diagnostic accuracy for respiratory tract infections.

The cut-off value in our research was 8.84 ng/ml. The mentioned studies showed heterogeneity in their cut-off values, which varied from 0.312 ng/mL to 118 ng/mL in the meta-analysis study [29]. This can be explained by using a different ELISA kit, using PTX3 from different sources, and using different case distributions.

There are several limitations to this study. Firstly, there are many techniques to evaluate pentraxin 3 in various body fluids. As a result, we advise measuring it in many settings and using different techniques for LRTIs, such as Bronchoalveolar lavage (BAL) in VAP and pleural fluid in PPE patients. Secondly, the number of subjects available for study was small, as we presented a single-center experience in one city in Egypt. Therefore, multicenter trials are advised. Thirdly, while we relied on routine culturing methods to identify bacterial pathogens, several additional pathogenic species, such as viruses, fungi, and atypical bacterial organisms, were not identified. Finally, Because of limited resources, the authors could not assess other biomarkers such as procalcitonin, D-dimer, and TNF-α to correlate with PTX3 levels. It is necessary to perform a more thorough investigation involving a greater number of patients and the levels of various biomarkers during the LRTIs.

## Conclusions

Serum PTX-3 is useful for accurately diagnosing LRTIs and predicting their severity and is a good prognostic biomarker. Specifically, serum PTX3 levels ≥ 8.84 ng/ml have been associated with greater sensitivity and NPV, which may allow prompt and precise identification of the great majority of actual negative cases.

## Data Availability

The datasets used during the current study are available from the corresponding author on reasonable request.

## Abbreviations

ABG: Arterial blood gas analysis
AUC: Area under curve
BAL: Bronchoalveolar lavage
CAP: Community-acquired pneumonia
CBC: Complete blood count
CDC: Centers for disease control and prevention
CoNS: Coagulase-negative staphylococci
CRP: C-reactive protein
ELISA: Enzyme-linked immunosorbent assay
ESR: Erythrocyte sedimentation rate
ICU: Intensive care unit
LRTIs: Lower respiratory tract infections
MRSA: Methicillin-resistant staphylococcus aureus
NPP: Negative predictive value
OD: Optical densities
PCT: Procalcitonin
PICU: Pediatric intensive care unit
PPE: Parapneumonic effusion
PPV: Positive predictive value
PRESS: Pediatric Respiratory Severity Score
PTX3: Pentraxin 3
ROC: Receiver Operating Characteristic
SD: Standard deviation
SPSS: Statistical Package of Social Science
TLC: Total Leucocytic Count
VAP: Ventilator-associated pneumonia
WHO: World Health Organization
